# Derivation of the small-angle scattering profile of a target biomacromolecule from a profile deteriorated by aggregates. AUC–SAS

**DOI:** 10.1107/S1600576723002406

**Published:** 2023-04-25

**Authors:** Ken Morishima, Rintaro Inoue, Masaaki Sugiyama

**Affiliations:** a Institute for Integrated Radiation and Nuclear Science, Kyoto University, 2-1010 Asashironishi, Kumatori, Sennan-gun, Osaka 590-0494, Japan; Australian Centre for Neutron Scattering, ANSTO, Australia

**Keywords:** small-angle X-ray scattering, small-angle neutron scattering, analytical ultracentrifugation, protein solutions, aggregates, biomacromolecules

## Abstract

Aggregates cause a fatal problem in the structural analysis of a biomacro­mol­ecule in solution using small-angle X-ray or neutron scattering (SAS): they deteriorate the scattering profile of the target molecule and lead to an incorrect structure. Recently, an integrated method of analytical ultracentrifugation (AUC) and SAS, abbreviated AUC–SAS, was developed as a new approach to overcome this problem. However, the original version of AUC–SAS does not offer a correct scattering profile of the target molecule when the weight fraction of aggregates is higher than *ca* 10%. In this study, the obstacle point in the original AUC–SAS approach is identified. The improved AUC–SAS method is then applicable to a solution with a relatively larger weight fraction of aggregates (≤20%).

## Abstract 2

An integrated method using analytical ultracentrifugation (AUC) and small-angle scattering (SAS), AUC–SAS, has been developed for the structural analysis of a biomacromolecule in solution. In this study, the first version of AUC–SAS is improved upon so as to be applicable to a solution with a large number of aggregates.

## 1. Introduction

Small-angle X-ray and neutron scattering (SAXS and SANS), collectively abbreviated as SAS, are increasingly being used to reveal structures of biomacromolecules in solution (Svergun & Koch, 2003 ▸; Bernadó *et al.*, 2018 ▸; Mahieu & Gabel, 2018 ▸). Modern computational analysis methods for SAS offer a detailed three-dimensional structural model (Grant, 2018 ▸; Bengtsen *et al.*, 2020 ▸; Gräwert & Svergun, 2020 ▸; Matsumoto *et al.*, 2020 ▸; Okuda *et al.*, 2021 ▸; Shimizu *et al.*, 2022 ▸; Yunoki *et al.*, 2022 ▸). To build a reliable structural model using these methods, it is crucial to obtain an experimental scattering profile that purely corresponds to the target molecule. However, even with a small content of aggregates (<10%), the scattering profile deteriorates from that of the target molecule and can result in an incorrect structural model. Moreover, there is another serious problem related to aggregates. Typically, an abnormal upturn of the scattering profile in the lowest scattering-angle region is recognized as experimental evidence of aggregate contamination. However, the scattering profile cannot show such clear evidence when the weight fraction of the aggregates is low. For example, the Guinier approximation holds for a sample with a small weight fraction of aggregates, and the scattering profile is expressed as a straight line in the Guinier plot, which gives the gyration radius of the sample biomacromolecule. However, when the gyration radius is larger than the expected radius, it is difficult to determine whether the solution includes aggregates or whether the target molecule itself is deformed from the expected structure. Accordingly, to solve the ‘aggregation problems’ of the identification and removal of aggregates, SAS coupled with other methods, such as size-exclusion chromatography (SEC–SAXS), has been explored (David & Pérez, 2009 ▸; Ryan *et al.*, 2018 ▸; Inoue *et al.*, 2019 ▸).

Recently, another integrated approach using analytical ultracentrifugation (AUC) and SAS, abbreviated AUC–SAS (Morishima *et al.*, 2020 ▸), has been developed to overcome aggregation problems. AUC–SAS derives a scattering profile of the target molecule in the solution including aggregates by utilizing the molecular distribution obtained with AUC. AUC–SAS reportedly offers precise scattering profiles of several bio­macromolecules in solution (Hirano *et al.*, 2021 ▸; Okuda *et al.*, 2021 ▸). Because AUC–SAS does not require a large amount of sample or a very high intensity instrument, as needed by synchrotron-light SAXS, it has the potential to be applied to laboratory-based SAXS. AUC–SAS is also applicable to SANS, which faces the same aggregation problem.

Improvement of AUC–SAS will expand the scope of wider applications. For example, the first version of AUC–SAS (‘first AUC–SAS’) was constrained by the weight fraction of the aggregates (less than ∼10%). In the present study, we have improved AUC–SAS, making it applicable to samples with relatively large weight fractions of aggregates (>10%). Furthermore, we provide software for the improved AUC–SAS, which is available to any SAS experimenter.

## 2. Experimental

### 2.1. Samples

Bovine serum albumin (BSA), apoferritin (AF), catalase (Cat), lysozyme (Lyz), ovalbumin (OVA) and ribonuclease A (RNaseA) were purchased from Merck, Sigma–Aldrich (Darmstadt, Germany). Human βB2-crystallin (βB2-cry) clone (consistent with NCBI sequence NM 000496) in a pET3a plasmid was obtained from Genscript (Piscataway, New Jersey, USA). The recombinant βB2-cry plasmid was then used to transform competent BL21(DE3)pLysS cells (Thermo Fisher Scientific, Waltham, Massachusetts, USA). Purification of βB2-cry was performed by following previous reports (Lampi *et al.*, 2006 ▸).

BSA, AF, Lyz, OVA and RNaseA were dissolved in 100 m*M* Tris–HCl buffer (pH 7.5) containing 100 m*M* NaCl. Cat and βB2-cry were dissolved in 50 m*M* Tris–HCl buffer (pH 8.0) containing 150 m*M* NaCl. The protein solutions were purified by SEC with a Superdex 200 Increase 10/300 GL column (for BSA, Cat, βB2-cry and OVA), Superose 6 Increase 10/300 GL column (for AF) and Superdex 75 Increase 10/300 GL column (for Lyz and RNaseA). The protein solutions were prepared by mixing the main component and its aggregate fractions while keeping the weight fraction of aggregates (*r*_a_) ≤ 0.2. Sample codes are expressed as [protein + number] (*e.g.* BSA6), where the number corresponds to *r*_a_. The mass concentrations for the AUC and SAXS measurements were 2.0 mg ml^−1^ for BSA6, BSA13, BSA20, AF5, AF15 and AF21; 2.1 mg ml^−1^ for Cat3 and Cat8; 1.1 mg ml^−1^ for Lyz6; 2.3 mg ml^−1^ for βB2-cry11; 2.2 mg ml^−1^ for OVA4; and 2.0 mg ml^−1^ for RNaseA8. BSA3 was subjected to AUC and SANS measurements after dialysis in D_2_O buffer.

### 2.2. AUC measurements

Sedimentation velocity AUC measurements were per­formed using ProteomeLab XL-I (Beckman Coulter, USA). The samples were loaded into cells equipped with 1.5 mm path length titanium center pieces (Nanolytics, Germany). All measurements were performed using Rayleigh interference optics at 298 K. The rotor speed was set at 45 000 r min^−1^ for BSA, AF, Cat, βB2-cry and OVA; and 60 000 r min^−1^ for Lyz and RNaseA. The time evolution of the sedimentation data was analyzed using the multi-component Lamm equation (Lebowitz *et al.*, 2002 ▸). The weight-concentration distribution *c*(*s*_20,w_) as a function of the sedimentation coefficient and frictional ratio *f*/*f*_0_ was computed using the *SEDFIT* software (version 15.01c) (Schuck, 2000 ▸). The sedimentation coefficient was normalized to be the value at 293 K in pure water, *s*_20,w_. The weight fraction of the *j*-mer, *r*_*j*_, was obtained from the corresponding peak area of *c*(*s*_20,w_). The molecular weight, *M*_*j*_, of the *j*-mer was calculated using the corresponding peak positions *s*_20,w,*j*_ and *f*/*f*_0_ (Brown & Schuck, 2006 ▸) as

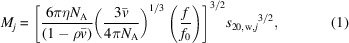

where η, ρ, *N*_A_ and 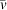 are the viscosity of water at 293 K, the density of water at 293 K, Avogadro’s number and the partial specific volume of the protein, respectively.

### 2.3. SAXS measurements

SAXS measurements were performed using a laboratory-based instrument (NANOPIX, Rigaku, Japan) equipped with a high-brilliance point-focused generator of a Cu *K*α source (MicroMAX-007 HFMR, Rigaku, Japan) (wavelength = 1.54 Å). Scattered X-rays were measured using a HyPix-6000 hybrid photon counting detector (Rigaku, Japan) composed of 765 × 813 pixels with a spatial resolution of 100 µm. For all samples, the sample-to-detector distance (SDD) was set to 1330 mm, with which the covered *q* range was 0.01 ≤ *q* ≤ 0.20 Å^−1^ (where *q* is the magnitude of the scattering vector). Two-dimensional scattering patterns were converted to one-dimensional scattering profiles using the *SAngler* software (Shimizu *et al.*, 2016 ▸). After correction by the transmittance and subtraction of buffer scattering, the absolute scattering intensity was obtained using the standard scattering intensity of water (1.632 × 10^−2^ cm^−1^) (Orthaber *et al.*, 2000 ▸). All measurements were performed at 298 K.

### 2.4. SEC–SAXS measurements

SEC–SAXS measurements were conducted with a laboratory-based SEC–SAXS system (La-SSS) (Inoue *et al.*, 2019 ▸), which is made up of a NANOPIX combined with a Prominence high-performance liquid chromatography system (SHIMADZU, Japan). A Superdex 200 Increase 10/300 GL for BSA, Cat, βB2-cry and OVA, a Superose 6 Increase 10/300 GL for AF, and a Superdex 75 Increase 10/300 GL for Lyz and RNaseA were utilized as the SEC column. All measurements were performed at a flow rate of 0.02 ml min^−1^ at 298 K.

### 2.5. SANS measurements

SANS measurements were performed using the SANS-U instrument located at JRR-3 (Japan Atomic Energy Agency, JAEA). A neutron beam at a wavelength of 6.0 Å with 10% resolution was irradiated on the samples. Scattered neutrons were counted using a two-dimensional detector (Ordela, USA). The SDDs were set to 4000 and 1030 mm, which covered a *q* range of 0.010–0.35 Å^−1^. Two-dimensional scattering patterns were converted to one-dimensional scattering profiles using the *Red2D* software (https://github.com/hurxl/Red2D). After correction by the transmittance and subtraction of buffer scattering, the absolute scattering intensity was obtained with the standard scattering intensity of H_2_O (0.89 cm^−1^) (Shibayama *et al.*, 2005 ▸). All measurements were performed at 298 K.

## 3. Methodology

In this section, we explain how to derive the scattering profile of a monomer from that of a solution that includes aggregates by the AUC–SAS method (see §1 of the supporting information for further details), and present the problems in applying the first AUC–SAS to a solution with a high weight fraction of aggregates.

### 3.1. Derivation of the scattering profile of protein monomer from an ensemble-averaged scattering profile

The scattering profile of the monomer and its aggregates, *I*(*q*), is represented as

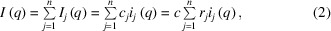

where *j* denotes the association number (1 ≤ *j* ≤ *n*); *I*_*j*_(*q*), *c*_*j*_ and *i*_*j*_(*q*) are the scattering profile, weight concentration and concentration-normalized scattering profile [*i*_*j*_(*q*) = *I*_*j*_(*q*)/*c*_*j*_] for the *j*-mer, respectively; and *c* and *r*_*j*_ are the total concentration ( 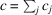 ) and weight fraction for the *j*-mer (*r*_*j*_ = *c*_*j*_/*c*), respectively. Since a *j*-mer could have diverse configurations, *I*_*j*_(*q*) indicates the ensemble-average scattering profile of all *j*-mers. Here, *c* is low, as the scattering profile is free from the interparticle interference effect.

To solve equation (2) for *I*_1_(*q*), the weight fractions of all components, {*r*_*j*_} (*j* ≥ 1) (#1), and the scattering profiles of aggregates, *i*_*j*_(*q*) (*j* ≥ 2) (#2), are required. As a prerequisite, highly denatured proteins and high-order aggregates are removed from the sample solution through the purification for a general SAS measurement. Hence, it is reasonable to assume that the residual aggregates are 4-mer at most (*j* ≤ 4) and that the total weight fraction of the aggregates, *r*_a_ (≡ 1 − *r*_1_), is <0.2. If this prerequisite is not satisfied (*i.e.* *j* > 4 and/or *r*_a_ > 0.2), the sample should be re-purified. Under these conditions, AUC offers information #1 ({*r*_*j*_}) (§2 of the supporting information). Next, to obtain information #2 [*i*_*j*_(*q*) (*j* ≥ 2)], we divided *i*_*j*_(*q*) into two *q* regions, *i*_*j*H_(*q*) and *i*_*j*L_(*q*), in the sufficiently high and lower *q* regions, respectively. Here, *i*_*j*H_(*q*) (*j* ≥ 2) could be identical to *i*_1H_(*q*) [*i*_*j*H_(*q*) ≃ *i*_1H_(*q*)] because there is no difference in the inner local structure between the monomer and the aggregates under the prerequisite conditions (no highly denatured aggregates in the sample). Therefore, *I*_1H_(*q*) is obtained using *I*(*q*) and *r*_1_ as follows (see §1 of the supporting information for further details):

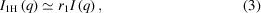

where *I*(*q*) and *r*_1_ are experimentally offered by SAS and AUC, respectively.

On the other hand, extrapolation of equation (3) to the lower *q* region, *i*_*j*L_(*q*) ≃ *i*_1L_(*q*), does not hold (open magenta circles in Fig. S1 of the supporting information.). Therefore, *I*_1L_(*q*) is considered as follows. First, the forward scattering intensity, *I*_1_(0), is obtained with *I*(0), *r*_*j*_ and *M*_*j*_, which are experimentally given by SAS and AUC, as follows (see §1 of the supporting information for further details): 

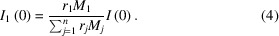

The remaining issue is a way to obtain *I*_1L_(*q*) (*q* > 0); namely, connecting between *I*_1H_(*q*) and *I*_1_(0). The first AUC–SAS (Morishima *et al.*, 2020 ▸) connects them with the Guinier formula:

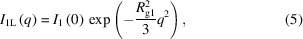

where *R*_g1_ is the gyration radius of the target monomer. As *R*_g1_ is an adjustable parameter, a reasonable *I*_1L_(*q*) is found by a smooth joint with *I*_1H_(*q*) at joint point *q*_c_. Finally, *I*_1_(*q*) is derived from *I*_1L_(*q*) (*q* ≤ *q*_c_) and *I*_1H_(*q*) (*q* > *q*_c_), and the appropriate *R*_g1_ is also provided (see §1 of the supporting information for further details).

### 3.2. Problems in first AUC–SAS

**Figure 1:**
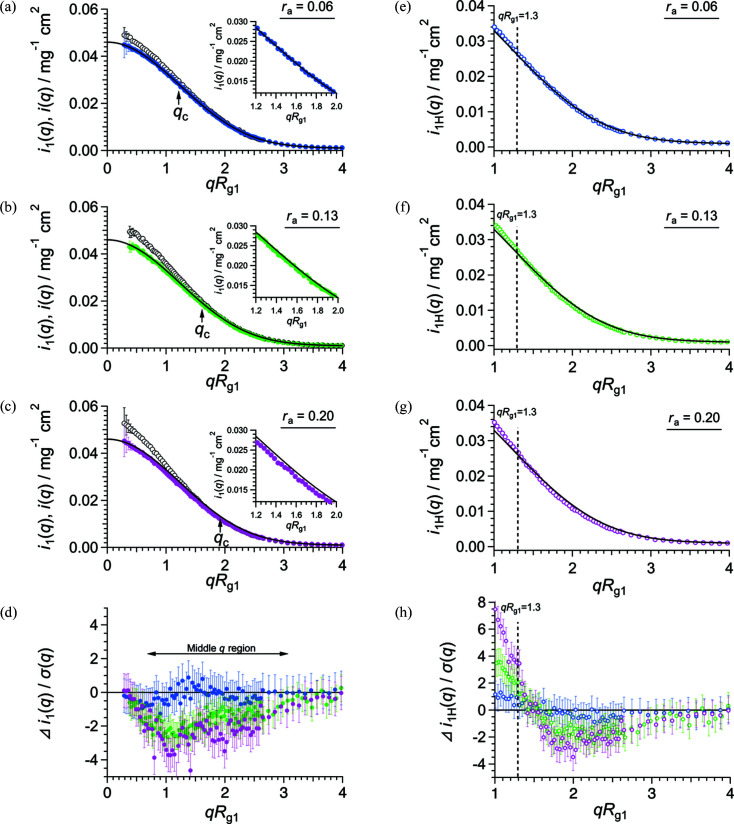
First AUC–SAS for the BSA solutions with various weight fractions of aggregates. (*a*)–(*c*) Filled blue, green and purple circles show *i*_1_(*q*) [= *I*_1_(*q*)/*c*_1_] which is derived by the first AUC–SAS for the BSA solutions with *r*_a_ = 0.06 (BSA6), 0.13 (BSA13) and 0.20 (BSA20), respectively. Open black circles show the experimental SAXS data *i*(*q*) [= *I*(*q*)/*c*]. Black lines represent *i*_1_(*q*)_Xtal_ calculated from the crystal structure of BSA monomer (PDB code 4f5s). Arrows indicate the connection points *q*_c_ between *i*_1L_(*q*) and *i*_1H_(*q*). Insets show the enlarged pictures in the range 1.2 ≤ *qR*_g1_ ≤ 2.0. (*d*) Filled blue, green and purple circles show the residuals Δ*i*_1_(*q*)/σ(*q*) for BSA6, BSA13 and BSA20, respectively. Here, Δ*i*_1_(*q*) = *i*_1_(*q*) − *i*_1_(*q*)_Xtal_ and σ(*q*) denotes the error of *i*_1_(*q*). (*e*)–(*g*) Open blue, green and purple circles show *i*_1H_(*q*) [= *I*_1H_(*q*)/*c*_1_] given by equation (3) for BSA6, BSA13 and BSA20, respectively. The black line in each panel represents *i*_1_(*q*)_Xtal_ calculated from the crystal structure of the BSA monomer. (*h*) Open blue, green and purple circles denote the residuals Δ*i*_1H_(*q*)/σ(*q*) for BSA6, BSA13 and BSA20, respectively. Here, Δ*i*_1H_(*q*) = *i*_1H_(*q*) − *i*_1_(*q*)_Xtal_ and σ(*q*) is the error of *i*_1H_(*q*). The broken line denotes the upper limit of the Guinier approximation range (*qR*_g1_ = 1.3).

Figs. 1 ▸(*a*)–1 ▸(*c*) show the concentration-normalized scattering profiles, *i*_1_(*q*) [= *I*_1_(*q*)/*c*_1_], that were derived with the first AUC–SAS. The samples were BSA solutions with different weight fractions of aggregates (*a*) *r*_a_ = 0.06, (*b*) 0.13 and (*c*) 0.20. The experimental AUC and SAXS data are shown in §2 of the supporting information and as open black circles in Figs. 1 ▸(*a*)–1 ▸(*c*), respectively. The black lines in Figs. 1 ▸(*a*)–1 ▸(*c*) represent the concentration-normalized scattering profiles, *i*_1_(*q*)_Xtal_, calculated from the crystal structure of the BSA monomer (PDB code 4f5s; Bujacz, 2012 ▸). Here, *i*_1_(*q*)_Xtal_ is identical to that obtained using SEC–SAXS for a BSA solution (Bucciarelli *et al.*, 2018 ▸). Fig. 1 ▸(*d*) shows the deviations between the scattering profile derived from the first AUC–SAS, *i*_1_(*q*), and that calculated from the crystal structure, *i*_1_(*q*)_Xtal_, *i.e.* Δ*i*_1_(*q*)/σ(*q*). Here, Δ*i*_1_(*q*) = *i*_1_(*q*) − *i*_1_(*q*)_Xtal_ and σ(*q*) is the error of *i*_1_(*q*). The first AUC–SAS successfully offered reasonable *i*_1_(*q*) at *r*_a_  = 0.06 [Δ*i*_1_(*q*)/σ(*q*) < 1] but produced a large deviation in the middle *q* region (0.5 ≤ *qR*_g1_ ≤ 3) at *r*_a_ = 0.13 and 0.20 [Δ*i*_1_(*q*)/σ(*q*) > 1]. As a result, *R*_g1_ at *r*_a_ = 0.06 (*R*_g1_ = 27.2 ± 0.2 Å) is consistent with that of the crystal structure (*R*_g1,Xtal_ = 27.1 Å), whereas the *R*_g1_s at *r*_a_ = 0.13 and 0.20 (*R*_g1_ = 27.5 ± 0.2 Å and *R*_g1_ = 28.1 ± 0.2 Å, respectively) are larger than *R*_g1,Xtal_ [*R*_g1_ and *i*_1_(0) are listed in Table S1 of the supporting information].

As shown in Figs. 1 ▸(*e*)–1 ▸(*h*), *i*_1H_(*q*) [= *I*_1H_(*q*)/*c*_1_], which is given by equation (3), deviated from *i*_1_(*q*)_Xtal_ even more in the higher *q* region than in the Guinier region (1.3 < *qR*_g1_ < 3) at *r*_a_ = 0.13 and 0.20. The large deviations, Δ*i*_1H_(*q*)/σ(*q*), at *r*_a_ = 0.13 and 0.20 in the middle *q* region make the connection points, *q*_c_, shift to the out-of-Guinier region (*q*_c_ *R*_g1_ ≃ 1.6 and 1.9, respectively). Consequently, incorrect *R*_g1_ and scattering profiles were obtained. To solve this problem, the connection should be performed in the Guinier region, that is, *I*_1H_(*q*) is correctly extrapolated to the inside of the Guinier region. In this study, we have developed a method to correctly extrapolate *I*_1H_(*q*) and offer a reasonable *I*_1_(*q*), even for relatively large *r*_a_.

## 4. Results and discussion

### 4.1. Scattering profile of aggregates

**Figure 2:**
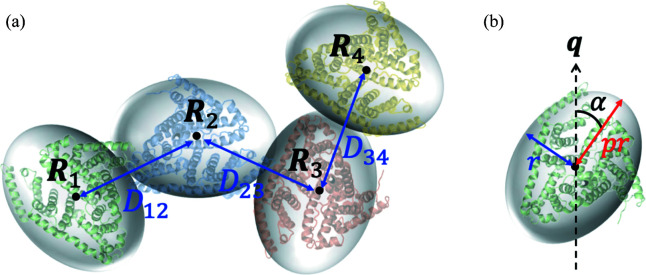
Schematic illustrations of an aggregate and a subunit. (*a*) A schematic illustration of an aggregate (*j* = 4) in which the subunits are randomly arranged. Black points and blue arrows represent the COMs of the subunits and the distances between the COMs of neighboring subunits, respectively. (*b*) A schematic illustration of the ellipsoidal approximation of a subunit. Blue and red arrows represent the semi-axes. The broken black arrow indicates the scattering vector.

The approximation of *i*_*j*H_(*q*) ≃ *i*_1H_(*q*), which gives *I*_1H_(*q*) [equation (3)], holds in the sufficiently high *q* region (*qR*_g1_ > 3), as shown in Figs. 1 ▸(*e*)–1 ▸(*h*). To derive the appropriate *I*_1H_(*q*) that is correctly extrapolated to the inside of the Guinier region, we carefully reconsidered the scattering profile of an aggregate. First, the concentration-normalized scattering profile of the *j*-mer, *i*_*j*_(*q*), is represented as follows:

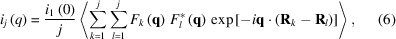

where **R**_*k*,*l*_ and *F*_*k*,*l*_(**q**) are the position vectors of the center of mass (COM) and the form factors of the *k*- or *l*-th subunit, respectively [Fig. 2 ▸(*a*)]. The form factor is normalized to be 〈|*F*_*k*,*l*_(0)|^2^〉 = 1, where 〈…〉 denotes the orientational average. The asterisk (*) denotes the complex conjugate.

Next, we assumed that the subunits were randomly arranged in the aggregate. According to the ‘decoupling approximation method’ (Kotlarchyk & Chen, 1983 ▸), the form factor is independent of the position in the aggregate: 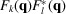 and exp[−*i* **q** · (**R**_*k*_ − **R**_*l*_)] in equation (6) can be decoupled, as in equation (S3) of the supporting information. Therefore, *i*_*j*_(*q*) can be expressed as follows (also see §4 of the supporting information):

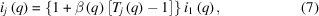

where

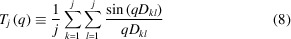

and

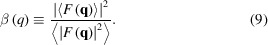


*T*_*j*_(*q*) is the inter-subunit structure factor defined by the Debye function [equation (8)] with the distance between the COMs of the *k*th and *l*th subunits, *D*_*kl*_. Considering the random arrangement of the subunits, *T*_*j*_(*q*) is expressed with the random flight model as equation (10). This model was originally developed for a synthetic polymer chain (Burchard & Kajiwara, 1970 ▸) and has been subsequently applied to randomly associated proteins (Larsen *et al.*, 2020 ▸).

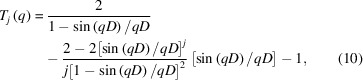

where *D* is the average distance between neighboring subunits (= 〈*D*_*k*,*k*+1_〉). Assuming that the gyration radius of a subunit, *R*_g1_, is the effective radius of the subunit, we defined *D* ≡ 2*R*_g1_ (see §5 of the supporting information).

β(*q*) indicates the shape anisotropy of the subunit [equation (9)]. Because the form factor of a subunit, *F*(**q**), is unknown prior to structural analysis of the monomer, we assumed that the subunit is an ellipsoid whose semi-axes are *r* and *pr* (*p* is the axial ratio), as shown in Fig. 2 ▸(*b*). Its form factor is then represented as follows:

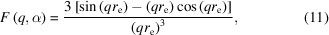

where

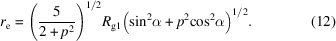

Then

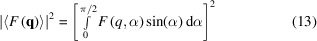

and

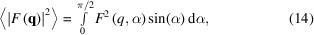

where α is the orientation angle between the axis of the ellipsoid and *q* [Fig. 2 ▸(*b*)]. β(*q*) was obtained by substituting equations (11)–(14) [Disp-formula fd13] [Disp-formula fd14] into equation (9). The axial ratio, *p*, is estimated using the frictional ratio *f*/*f*_0_, which is offered by the AUC measurement (Lebowitz *et al.*, 2002 ▸) (see further details in §6 of the supporting information).

### 4.2. Improved AUC–SAS

By substituting equation (7) into equation (2), *I*_1_(*q*) is expressed as follows:

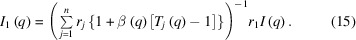

For this improvement, *I*_1H_(*q*) was calculated using equation (15), instead of equation (3). To estimate *R*_g1_ in *T*_*j*_(*q*) and β(*q*) [equations (10)–(14) [Disp-formula fd12] [Disp-formula fd13] [Disp-formula fd14]], the first AUC–SAS was initially used.

**Figure 3:**
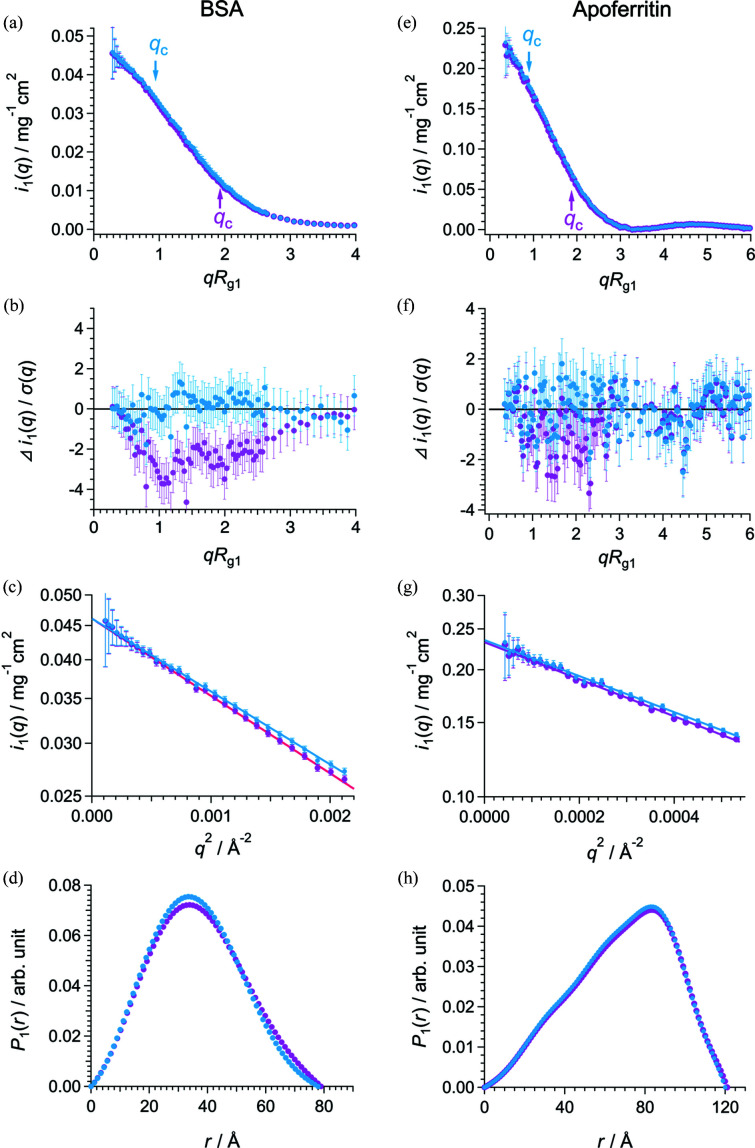
Demonstration of the first and improved AUC–SAS for BSA20 and AF21. In all panels, purple and cyan circles represent the results of the first AUC–SAS and improved AUC–SAS, respectively. Concentration-normalized scattering profiles, *i*_1_(*q*), for (*a*) BSA monomer and (*e*) AF 24-mer. Residuals Δ*i*_1_(*q*)/σ(*q*) for (*b*) BSA20 and (*f*) AF21. Guinier plots of *i*_1_(*q*) for (*c*) BSA20 and (*g*) AF21. Solid purple and cyan lines express the least-squares fitting lines with the Guinier formula. Pair distance distribution functions, *P*_1_(*r*), for (*d*) BSA20 and (*h*) AF21.

The improved method was demonstrated for BSA and AF solutions with *r*_a_ = 0.20 (BSA20) and *r*_a_ = 0.21 (AF21), respectively. Their experimental AUC data are shown in §2 of the supporting information. Fig. 3 ▸(*a*) shows *i*_1_(*q*) [= *I*_1_(*q*)/*c*_1_] which was derived using the first AUC–SAS (purple circles) and improved AUC–SAS (cyan circles) for BSA20. As shown in Fig. 3 ▸(*b*), the deviations Δ*i*_1_(*q*)/σ(*q*) for the improved AUC–SAS were sufficiently small [Δ*i*_1_(*q*)/σ(*q*) < 1] in the entire *q* region. As shown in Figs. 3 ▸(*c*) and 3 ▸(*d*) and Table 1 ▸, the improved AUC–SAS yielded more reasonable structural parameters [*R*_g1_, *i*_1_(0), *P*_1_(*r*) (pair distance distribution function) and *D*_max_] than the first AUC–SAXS. For the larger protein, AF solution (AF21), the improved AUC–SAS successfully gave reasonable *i*_1_(*q*) and structural parameters [Figs. 3 ▸(*e*)–3 ▸(*h*) and Table 1 ▸]. Thus, the improved AUC–SAS was applicable to a solution with a relatively large *r*_a_ (≤ 0.2), which is the general condition for most SAS measurements.

**Figure 4:**
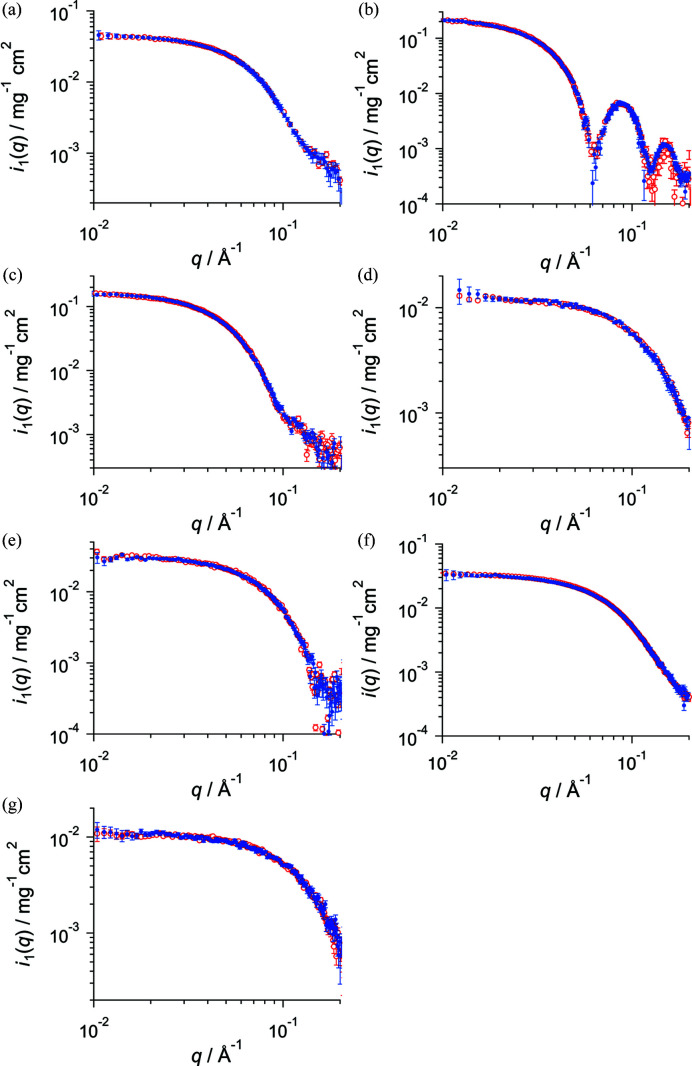
Open red and filled blue circles show the scattering profile *i*_1_(*q*) given by SEC–SAXS and improved AUC–SAS, respectively, for (*a*) BSA20, (*b*) AF21, (*c*) Cat8, (*d*) Lyz6, (*e*) βB2-cry11, (*f*) OVA4 and (*g*) RNaseA8.

Furthermore, we demonstrated the improved AUC–SAS for various proteins with different shapes and sizes (AUC results of the samples are shown in §2 of the supporting information). As shown in Fig. 4 ▸ and Table 2 ▸, the scattering profiles *i*_1_(*q*) and structural parameters [*R*_g1_ and *i*_1_(0)] offered by the improved AUC–SAS are consistent with those of SEC–SAXS for these proteins at various *r*_a_ (≤ 0.2).

AUC–SAS is applicable to SANS, which faces the same aggregation problem, as well as SAXS. We examined the AUC–SANS for a BSA solution (BSA3) using the improved AUC–SAS (§7 of the supporting information). For the SANS data of BSA3, the improved AUC–SAS successfully offered a reasonable scattering profile and gyration radius (*R*_g1_ = 26.5 ± 0.2 Å) that were consistent with those of the crystal structure (*R*_g1,Xtal_ = 26.7 Å). For neutron facilities without a SEC–SANS system (Jordan *et al.*, 2016 ▸; Johansen *et al.*, 2018 ▸; Sato *et al.*, 2021 ▸), AUC–SANS is the most promising method for obtaining the aggregation-free scattering profile.

In §8 and §9 of the supporting information, we evaluate the maximum errors originated by the random flight model and ellipsoidal approximation. The error in *I*_1_(*q*) is several per cent at most, even though the extreme cases are assumed.

It is often worthwhile analyzing the structure of the aggregate (Kovalchuk *et al.*, 2019 ▸). Programs such as *SASREFMX* and *OLIGOMER* in the *ATSAS* package (Petoukhov *et al.*, 2012 ▸; Manalastas-Cantos *et al.*, 2021 ▸) are well known for modeling of aggregates. However, these programs require the structure of the monomer. Hence, the complementary use of AUC–SAS and these programs is a promising strategy.

Implementing the improved AUC–SAS, *Igor Pro*-based software (Kline, 2006 ▸) has been developed for the utilization of AUC–SAS by SAS experimenters. The required information is the data set of molecular weights (or association number), weight fractions and the frictional ratio, which are given by AUC. The scattering profile of the target monomer is obtained just by inputting the AUC information and SAS profile for the solution. The software is available at https://www.rri.kyoto-u.ac.jp/NSBNG/activity.html. Its usage is described in §10 of the supporting information.

## 5. Related literature

The following additional references are only cited in the supporting information for this article: Perkins (2001 ▸), Perrin (1934 ▸), Pierce *et al.* (2014 ▸).

## Supplementary Material

Supporting information. DOI: 10.1107/S1600576723002406/ge5127sup1.pdf

## Figures and Tables

**Table 1:** Gyration radii, forward scattering intensities, molecular weights calculated from forward scattering intensities, and maximum pair distances for BSA20 and AF21 *R*_g_ and *i*(0): gyration radius and concentration-normalized forward scattering intensity for non-treated SAXS, respectively. *R*_g1_ and *i*_1_(0): gyration radius and concentration-normalized forward scattering intensity of the monomer, respectively, which were derived using AUC–SAS. *M*: molecular weight calculated from the forward scattering intensity. *D*_max_: maximum pair distance from *P*_1_(*r*). The error of the gyration radius is the standard deviation. The errors of the concentration-normalized forward scattering intensity and molecular weight were calculated from the standard deviations of the forward scattering intensity and concentration.

	*R*_g_, *R*_g1_ (Å)	*i*(0), *i*_1_(0) (mg^−1^cm^2^)	*M* (kDa)	*D*_max_
BSA20				
Non-treated SAXS	30.9 ± 0.3	0.0565 ± 0.0005	84.2 ± 0.7	98.5
First AUC–SAS	28.1 ± 0.2	0.0461 ± 0.0004	68.7 ± 0.6	78.8
Improved AUC–SAS	27.3 ± 0.2	0.0468 ± 0.0004	69.6 ± 0.6	77.9
Crystal structure†	27.1	0.0465	69.2	77.0

AF21				
Non-treated SAXS	61.5 ± 0.8	0.280 ± 0.010	604 ± 20	175
First AUC–SAS	55.1 ± 0.5	0.235 ± 0.009	507 ± 19	122
Improved AUC–SAS	54.1 ± 0.5	0.239 ± 0.009	516 ± 19	120
Crystal structure‡	54.0	0.221	477	119

† PDB code 4f5s.

‡ PDB code 4v1w; Russo & Passmore (2014 ▸).

**Table 2:** Gyration radii and forward scattering intensities given by AUC–SAXS (improved AUC–SAXS) and SEC–SAXS for various proteins *R*_g1_ and *i*_1_(0): gyration radius and concentration-normalized forward scattering intensity, respectively. The error of the gyration radius is the standard deviation. The error of the concentration-normalized forward scattering intensity was calculated from the standard deviations of the forward scattering intensity and concentration.

Protein	Method	Sample code	*R*_g1_ (Å)	*i*_1_(0) (mg^−1^cm^2^)
BSA	Improved AUC–SAXS	BSA6 (*r*_a_ = 0.06)	27.2 ± 0.2	0.0461 ± 0.0006
		BSA13 (*r*_a_ = 0.13)	27.3 ± 0.2	0.0460 ± 0.0005
		BSA20 (*r*_a_ = 0.20)	27.3 ± 0.2	0.0468 ± 0.0004
	SEC–SAXS	–	27.2 ± 0.3	0.0455 ± 0.0019
AF	Improved AUC–SAXS	AF5 (*r*_a_ = 0.05)	54.4 ± 0.6	0.249 ± 0.008
		AF15 (*r*_a_ = 0.15)	54.3 ± 0.5	0.243 ± 0.009
		AF21 (*r*_a_ = 0.21)	54.1 ± 0.5	0.239 ± 0.009
	SEC–SAXS	–	53.9 ± 0.6	0.241 ± 0.015
Catalase	Improved AUC–SAXS	Cat3 (*r*_a_ = 0.03)	37.2 ± 0.2	0.155 ± 0.006
		Cat8 (*r*_a_ = 0.08)	37.3 ± 0.2	0.153 ± 0.005
	SEC–SAXS	Cat8 (*r*_a_ = 0.08)	37.1 ± 0.2	0.160 ± 0.008
Lyz	Improved AUC–SAXS	Lyz6 (*r*_a_ = 0.06)	15.0 ± 0.2	0.0125 ± 0.0008
	SEC–SAXS	–	15.1 ± 0.1	0.0124 ± 0.0012
βB2-cry	Improved AUC–SAXS	βB2-cry11 (*r*_a_ = 0.11)	22.6 ± 0.2	0.0319 ± 0.0007
	SEC–SAXS	–	22.7 ± 0.3	0.0325 ± 0.0013
OVA	Improved AUC–SAXS	OVA4 (*r*_a_ = 0.04)	23.9 ± 0.2	0.0346 ± 0.0005
	SEC–SAXS	–	23.9 ± 0.4	0.0340 ± 0.0009
RNaseA	Improved AUC–SAXS	RNaseA8 (*r*_a_ = 0.08)	15.0 ± 0.2	0.0110 ± 0.0004
	SEC–SAXS	–	14.8 ± 0.2	0.0116 ± 0.0003
