# Supplementary material for: Derivation of the small-angle scattering profile of a target biomacromolecule from a profile deteriorated by aggregates. AUC–SAS
Source: J Appl Crystallogr. 2023 Apr 25;56(Pt 3):624–32. doi: 10.1107/S1600576723002406 (PMC10241049; doi:10.1107/S1600576723002406)
Supplement: Supplementary file 1 [file j-56-00624-sup1.pdf]

JOURNAL OF
APPLIED
CRYSTALLOGRAPHY

Volume 56 (2023)

Supporting information for article:

Derivation of the small-angle scattering profile of a target biomacromolecule from the profile deteriorated by aggregates. AUC–SAS

Ken Morishima, Rintaro Inoue and Masaaki Sugiyama

SUPPLEMENTAL INFORMATION

Derivation of small-angle scattering profile of a target biomacromolecule from the profile deteriorated by aggregates: AUC-SAS

Ken Morishima, Rintaro Inoue, and Masaaki Sugiyama*

Institute for Integrated Radiation and Nuclear Science, Kyoto University, 2-1010 Asashironishi, Kumatori, Sennan-gun, Osaka, 590-0494, Japan

*To whom all correspondence should be addressed.
E-mail: sugiyama.masaaki.5n@kyoto-u.ac.jp

§1. Procedure of the first-AUC-SAS.

- **Step 1. AUC measurement.**

The weight fraction distribution $c(s_{20,\mathrm{w}})$ is obtained with AUC measurement (Figure S1(a)). The sedimentation coefficients $\{s_{20,\mathrm{w},j}\}$ molecular weights $\{M_j\}$ and weight fractions $\{r_j\}$ of j-mer are given from $c(s_{20,\mathrm{w}})$.

- **Step 2. SAS measurement.**

The scattering profile $I(q)$ is obtained with SAS measurement for the same solution with AUC measurement (black circles in Figure S1(b)). The gyration radius R_g and forward scattering intensity $I(0)$ are offered by Guinier analysis.

- **Step 3. Calculation of scattering intensity of monomer in high q-region, $I_{1\mathrm{H}}(q)$.**

As a prerequisite, highly denatured and aggregated proteins are removed from the sample solution by a SEC purification for a general SAS measurement. Hence, the inner local structure of j-mer is not different from that of monomer. In this situation, the concentration-normalized scattering intensity of j-mer in high q-region $i_{j\mathrm{H}}(q)$ are assumed to be identical with that of monomer, $i_{1\mathrm{H}}(q)$.

$$i_{j\mathrm{H}}(q) \cong i_{1\mathrm{H}}(q) \quad \text{(S1)}$$

Substituting Eq.(S1) into Eq.(2), we obtain Eq.(3).

$$I(q) = \sum_{j=1}^{n} I_j(q) = \sum_{j=1}^{n} c_j i_j(q) = c \sum_{j=1}^{n} r_j i_j(q), \quad (2)$$

$$I_{1\mathrm{H}}(q) \cong r_1 I(q)\,, \quad (3)$$

Utilizing r_1 and $I(q)$ which are experimentally given by AUC (Step 1) and SAS (Step 2), respectively, $I_{1\mathrm{H}}(q)$ is provided as the closed magenta circles in Figure S1(b). (Here, open magenta circles in Figure S1(b) show the extrapolation of $I_{1\mathrm{H}}(q)$ toward the lower q-region. Since Eq.(S1) does not hold in the lower q-region, Eq.(3) does not applicable in the lower q-region.)

- **Step 4. Calculation of forward scattering intensity of monomer, $I_1(0)$.**

Since the concentration-normalized forward scattering intensity is proportional to the molecular weight, the following equation is established.

$$i_j(0) = \frac{M_j i_1(0)}{M_1} \quad \text{(S2)}$$

Substituting Eq.(S2) into Eq.(2), the following equation is given:

$$I_1(0) = \frac{r_1 M_1}{\sum_{j=1}^{n} r_j M_j} I(0)\,. \quad (4)$$

Therefore, $I_1(0)$ is obtained with r_j, M_j, and $I(0)$ which are experimentally given by AUC (Step 1) and SAS (Step 2) as closed black square in Figure S1(b).

- **Step 5. Connection between $I_1(0)$ and $I_{1\mathrm{H}}(q)$ with Guinier formula.**

Since Guinier approximation should hold for the target monomer, the scattering profile of monomer in low q-region, $I_{1\mathrm{L}}(q)$, is given as Guinier formula.

$$I_{1L}(q) = I_1(0)\exp\left(-R_{g1}^2 q^2/3\right), \quad (5)$$

where $I_1(0)$ is determined with Eq.(4). $I_{1L}(q)$ is obtained by finding the appropriate gyration radius of monomer, R_{g1} as follows:

1. A candidate $I_{1L}(q)$ $(= I_{1L}^*(q)$; broken blue lines in Figure S1(c)) is calculated with Eq. (5) using a gyration radius R_{g1}^*.
2. $I_{1L}^*(q)$ and $I_{1H}(q)$ (broken blue line and open magenta circles in Figure S1(c), respectively) are jointed at the joint point q_c^* (open blue circles in Figure S1(c)).
3. The slopes of $I_{1L}^*(q)$ and $I_{1H}(q)$ at q_c^* (i.e., $\mathrm{d}I_{1L}^*(q_c^*)/\mathrm{d}q$ and $\mathrm{d}I_{1H}(q_c^*)/\mathrm{d}q$, respectively) are calculated.
4. With changing R_{g1}^*, the appropriate R_{g1} is found to realize $\mathrm{d}I_{1L}^*(q_c)/\mathrm{d}q = \mathrm{d}I_{1H}(q_c)/\mathrm{d}q$ (in other words, smooth joint of $I_{1L}^*(q)$ and $I_{1H}(q)$ at q_c).
5. $I_{1L}(q)$ (solid blue line in Figure S1(c)) is derived with the appropriate R_{g1}.

Finally, $I_1(q)$ is completed by $I_{1L}(q)$ $(q \leq q_c)$ and $I_{1H}(q)$ $(q > q_c)$.

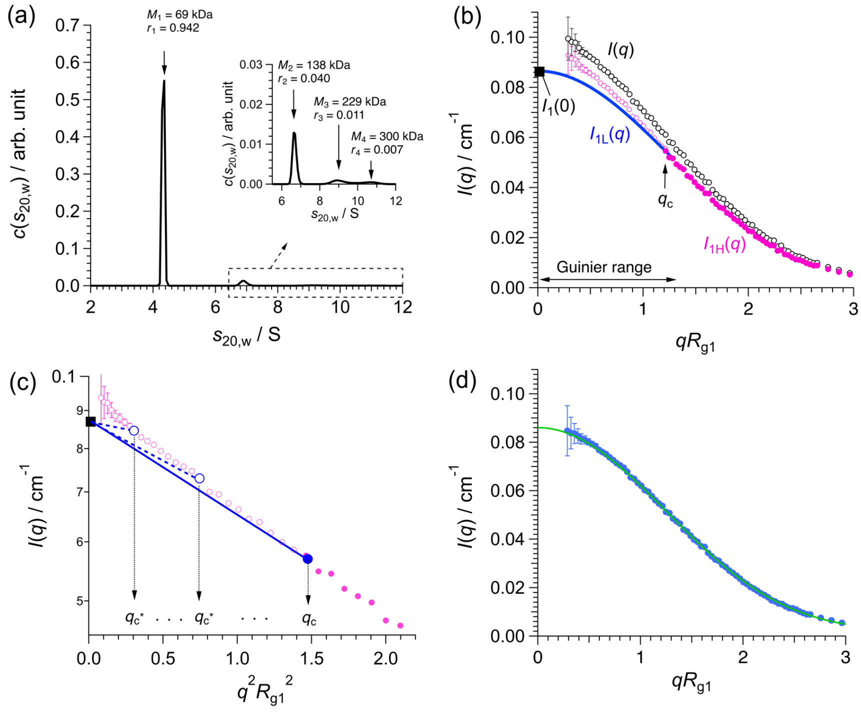

Figure S1. Demonstration of the first-AUC-SAS for BSA6: (a) Solid line shows $c(s_{20,\mathrm{w}})$ obtained by AUC. (b) Open black circles show the experimental SAXS profile, $I(q)$. Closed magenta circles, closed black square, solid blue line represent $I_{1H}(q)$, $I_1(0)$, and $I_{1L}(q)$, respectively. Open magenta circles show the extrapolation of $I_{1H}(q)$ toward the lower q-region. (c) Closed and open magenta circles represent $I_{1H}(q)$ and its extrapolation toward the lower q-region. Closed black square is $I_1(0)$. Broken blue lines and open blue circles show the candidates $I_{1L}^*(q)$ and q_c^*, respectively. Solid blue line and closed blue circle show the reasonable $I_{1L}(q)$ and q_c, where $\mathrm{d}I_{1L}(q_c)/\mathrm{d}q = \mathrm{d}I_{1H}(q_c)/\mathrm{d}q$ holds. (d) Closed blue circles show the finally derived $I_1(q)$. The green line shows the scattering profile calculated from the crystal structure (PDB code: 4F5S).

§2. AUC data for protein solutions with various weight fractions of aggregates.

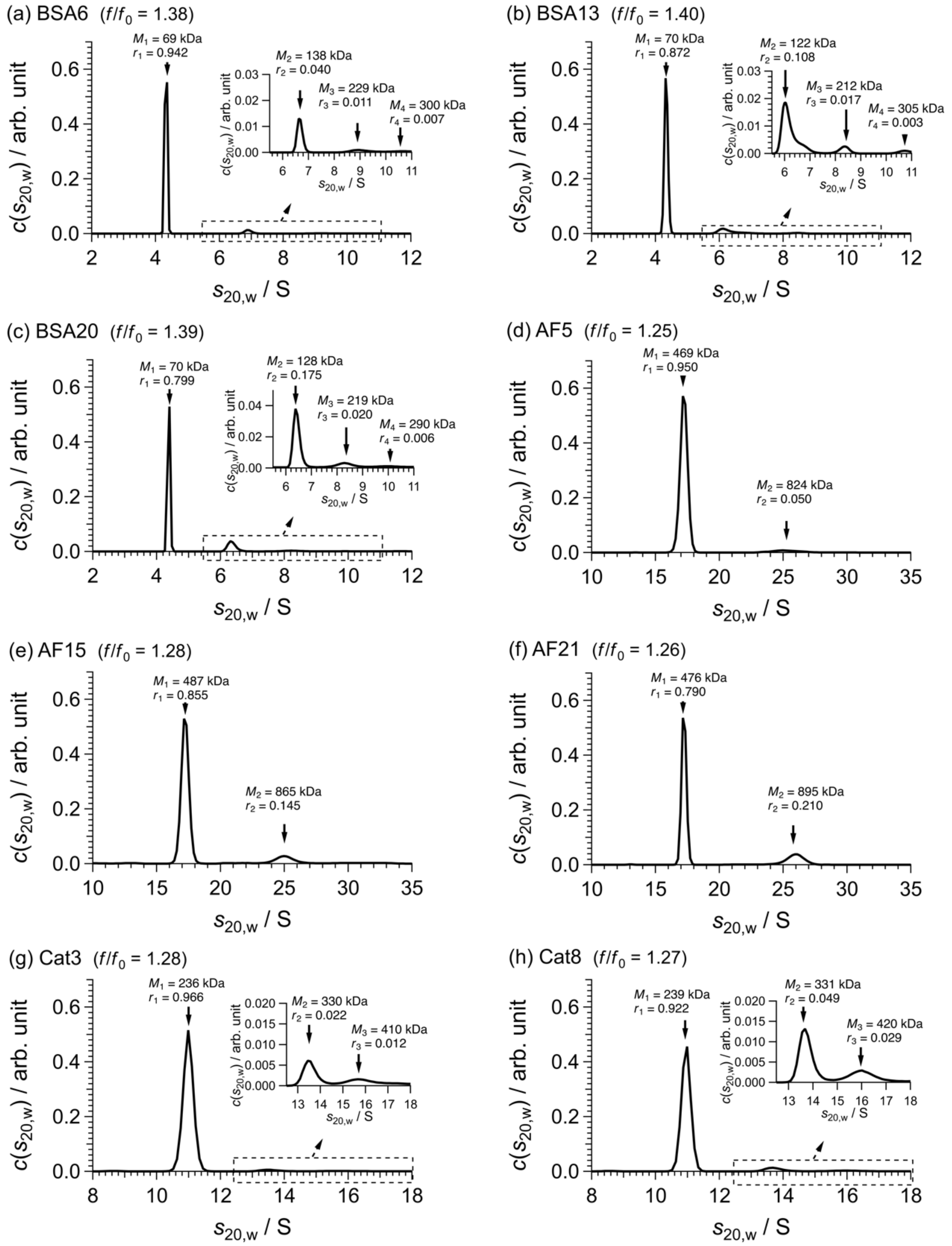

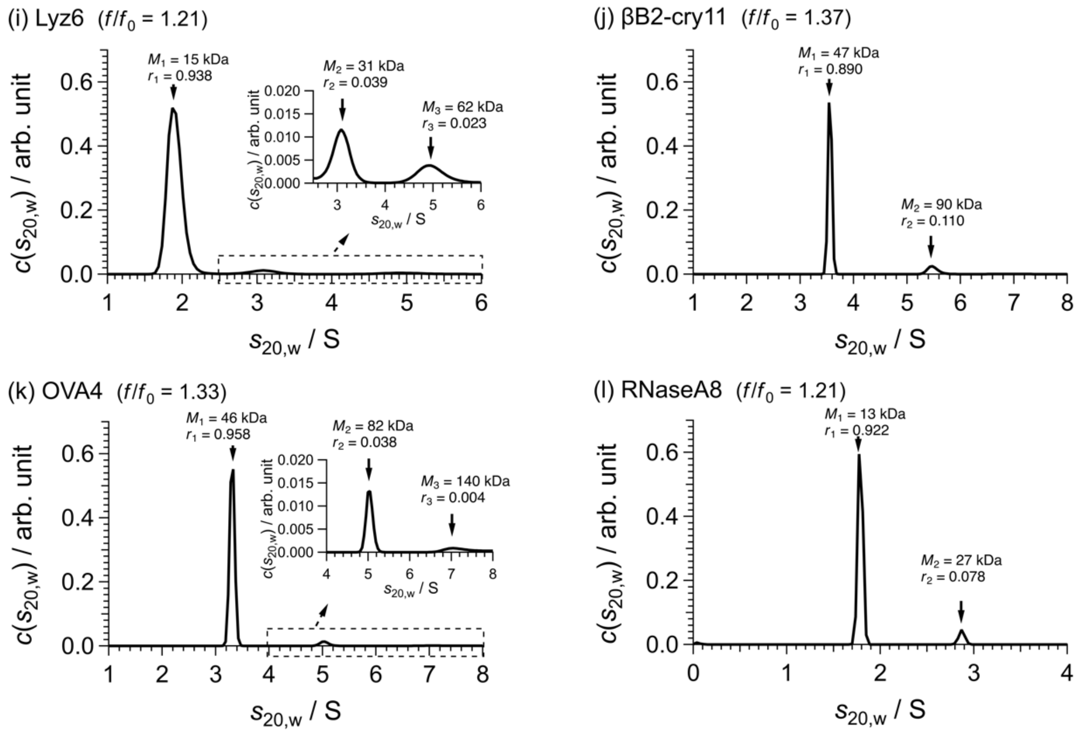

Figure S2. Weight concentration distributions, $c(s_{20,\mathrm{w}})$, obtained with AUC for BSA6 (a), BSA13 (b), BSA20 (c), AF5(d), AF15(e), AF21(f), Cat3(g), Cat8(h), Lyz6(i), βB2-cry11(j), OVA4(k), RNaseA8(l). Molecular weights M_j, weight fractions r_j, and frictional ratio f/f_0 are represented in the figures.

§3. First-AUC-SAS for the solutions with various weight fractions of aggregates.

Table S1. Gyration radii, forward scattering intensities, and molecular weights calculated from forward scattering intensities for BSA6, BSA13, and BSA20

	R_g, R_{g1}/ Å	$i(0), i_1(0)$ / $mg^{-1}cm^2$	M / kDa
Non-treated SAXS (BSA6)	28.1 ± 0.2	0.0500 ± 0.0006	74.5 ± 0.9
First-AUC-SAS (BSA6)	27.2 ± 0.2	0.0461 ± 0.0006	68.7 ± 0.8
Non-treated SAXS (BSA13)	29.1± 0.2	0.0527 ± 0.0005	78.5 ± 0.7
First-AUC-SAS (BSA13)	27.5 ± 0.2	0.0460 ± 0.0005	68.6 ± 0.6
Non-treated SAXS (BSA20)	30.9 ± 0.3	0.0565 ± 0.0006	84.2 ± 0.8
First-AUC-SAS (BSA20)	28.1 ± 0.2	0.0461 ± 0.0005	68.7 ± 0.7
Crystal structure (PDB code: 4F5S)	27.1	0.0465	69.2

R_g and $i(0)$: gyration radius and concentration-normalized forward scattering intensity for non-treated SAXS, respectively. R_{g1} and $i_1(0)$: gyration radius and concentration-normalized forward scattering intensity of the monomer, respectively, derived using AUC-SAS. M: molecular weight calculated from the forward scattering intensity. The error of gyration radius is the standard deviation. The errors of concentration-normalized forward scattering intensity and molecular weight were calculated with the standard deviations of forward scattering intensity and concentration.

§4. Scattering profile of aggregates.

We assumed that the subunits were randomly arranged in aggregates. Based on the "decoupling approximation method" proposed by Kotlarchyk *et al.* (Kotlarchyk & Chen, 1983), the form factor ($F_m(\boldsymbol{q})$) is independent of the position ($\boldsymbol{R_m}$) of the aggregate: $F_k(\boldsymbol{q})F_l^*(\boldsymbol{q})$ and $\exp[-i\boldsymbol{q}\cdot(\boldsymbol{R_k}-\boldsymbol{R_l})]$ in Eq.(6) can be decoupled, as in Eq. (S3).

$$i_j(q)=\frac{i_1(0)}{j}\langle\sum_{k=1}^{j}\sum_{l=1}^{j}\langle F_k(\boldsymbol{q})F_l^*(\boldsymbol{q})\rangle\exp[-i\boldsymbol{q}\cdot(\boldsymbol{R_k}-\boldsymbol{R_l})]\rangle\ . \qquad \text{(S3)}$$

The inner bracket can be written as:

$$\langle F_k(\boldsymbol{q})F_l^*(\boldsymbol{q})\rangle=[\langle|F(\boldsymbol{q})|^2\rangle-|\langle F(\boldsymbol{q})\rangle|^2]\delta_{kl}+|\langle F(\boldsymbol{q})\rangle|^2\ . \qquad \text{(S4)}$$

Therefore, $i_j(q)$ is simplified in the following:

$$\begin{aligned}i_j(q)&=\frac{i_1(0)}{j}\langle\sum_{k=1}^{j}\sum_{l=1}^{j}\langle|F(\boldsymbol{q})|^2\rangle-|\langle F(\boldsymbol{q})\rangle|^2+|\langle F(\boldsymbol{q})\rangle|^2\exp[-i\boldsymbol{q}\cdot(\boldsymbol{R_k}-\boldsymbol{R_l})]\rangle\\&=i_1(0)\left[\langle|F(\boldsymbol{q})|^2\rangle-|\langle F(\boldsymbol{q})\rangle|^2+|\langle F(\boldsymbol{q})\rangle|^2T_j(q)\right]\\&=i_1(0)\left\{\langle|F(\boldsymbol{q})|^2\rangle+|\langle F(\boldsymbol{q})\rangle|^2\left[T_j(q)-1\right]\right\}\\&=\left\{1+\beta(q)\left[T_j(q)-1\right]\right\}i_1(q)\ ,\end{aligned} \qquad \text{(S5)}$$

where $T_j(q)$ and $\beta(q)$ are defined as follows:

$$T_j(q)\equiv\frac{1}{j}\sum_{k=1}^{j}\sum_{l=1}^{j}\frac{\sin(qD_{kl})}{qD_{kl}}\ , \qquad \text{(8)}$$

$$\beta(q)\equiv\frac{|\langle F(\boldsymbol{q})\rangle|^2}{\langle|F(\boldsymbol{q})|^2\rangle}\ . \qquad \text{(9)}$$

§5. Distance between neighboring subunits (*D*).

To define the distance between neighboring subunits D, the concentration-normalized scattering profile of the j-mer, $i_j(q)$, was calculated for various proteins (BSA, ovalbumin, and lysozyme) using Eq. (7), (9), and (10) in the two cases, $i_j(q)_{\mathrm{cal1}}$ with $D \equiv 2R_{\mathrm{g1}}$ and $i_j(q)_{\mathrm{cal2}}$ with $D \equiv 2(5/3)^{0.5}R_{\mathrm{g1}}$ which are the blue and red lines in Figure S3, respectively. The concentration-normalized scattering profile of the monomer, $i_1(q)$, in Eq. (7) was calculated from their crystal structures (PDB codes: 4F5S for BSA, 1OVA for ovalbumin, and 1LYZ for lysozyme).

As a reference, $i_j(q)$ was computed from the oligomer built by docking simulation (ZDOCK; Pierce *et al.*, 2014). The crystal structures of the monomers were used as subunits consisting of aggregates. We adopted the top ten oligomers in the docking score from the candidates offered by ZDOCK. The thick gray line in Figure S3 indicates the average $i_j(q)$ of the top ten oligomers (we defined this profile as $i_j(q)_{\mathrm{Dock}}$).

As shown in Figure S3, $i_j(q)_{\mathrm{cal1}}$ (blue line) better reproduces $i_j(q)_{\mathrm{Dock}}$ (gray line) than $i_j(q)_{\mathrm{cal2}}$ (red line) for all proteins. Thus, we adopted $D \equiv 2R_{\mathrm{g1}}$ as D in the random-flight model.

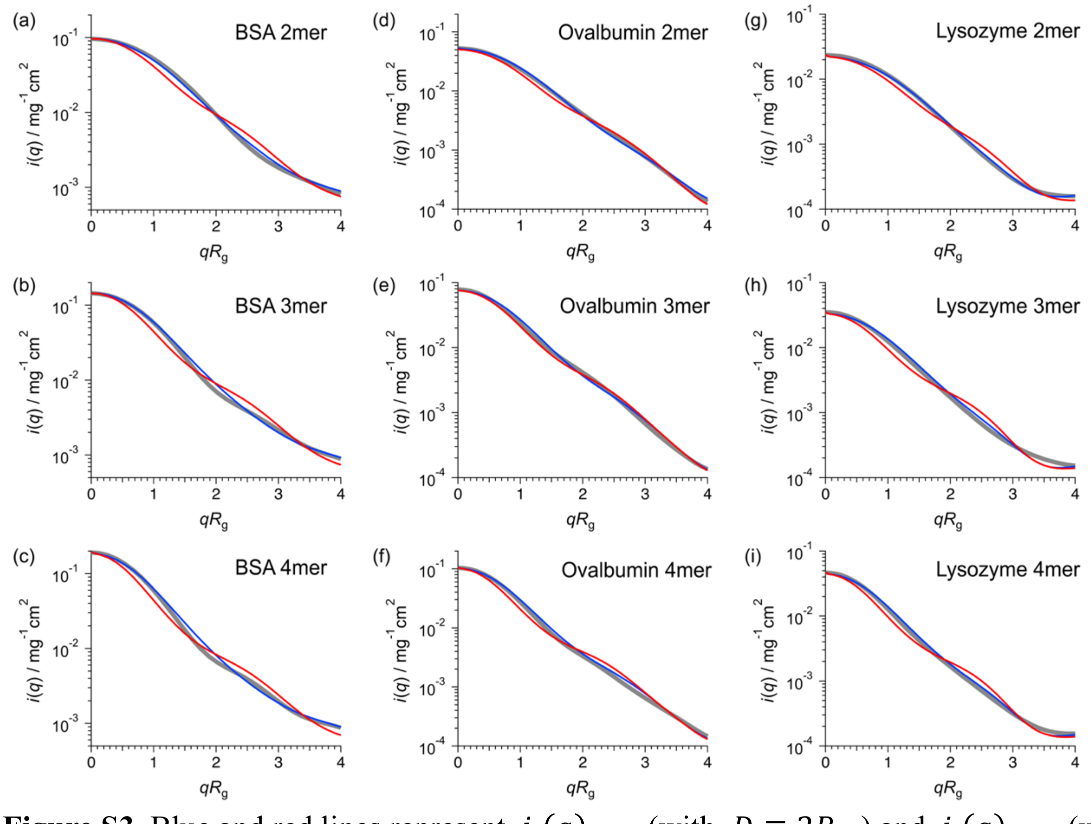

Figure S3. Blue and red lines represent $i_j(q)_{\mathrm{cal1}}$ (with $D \equiv 2R_{\mathrm{g1}}$) and $i_j(q)_{\mathrm{cal2}}$ (with $D \equiv 2(5/3)^{0.5}R_{\mathrm{g1}}$) which were calculated with random-flight model, respectively. Thick gray lines are $i_j(q)_{\mathrm{Dock}}$ calculated from the oligomer which was given by ZDOCK.

§6. Axial ratio (p) calculated from the frictional ratio (f/f_0).

The axial ratio p, of the ellipsoid is estimated using the frictional ratio f/f_0, which is given by the AUC measurement as follows:

As represented in Eq. (S6), f/f_0 is decomposed into two components: the contribution of shape asymmetry f/f_{sp}, and hydration f_{sp}/f_0 (Lebowitz *et al.*, 2002). Here, f is the frictional coefficient of the target particle, f_0 is the frictional coefficient of the spherical particle with the same molecular weight and density as the target particle, f_{sp} is the frictional coefficient of the spherical particle considering hydration.

$$\frac{f}{f_0} = \frac{f}{f_{\mathrm{sp}}}\frac{f_{\mathrm{sp}}}{f_0} = \frac{f}{f_{\mathrm{sp}}}\left(\frac{\bar{v}_2 + \delta\bar{v}_1}{\bar{v}_2}\right)^{\frac{1}{3}}, \qquad \text{(S6)}$$

where $\bar{v}_1$ and $\bar{v}_2$ are the partial specific volumes of the solvent and target particles, respectively. δ is the hydration of the particle (in grams of water per gram of the particle), for which a consensus value is commonly taken to be 0.3 g/g (Perkins, 2001).

The frictional ratio of shape asymmetry, f/f_{sp}, is related to the axial ratio, p, as follows (Perrin, 1934):

For prolate:

$$\frac{f}{f_{\mathrm{sp}}} = \frac{p^{-1/3}(p^2-1)^{1/2}}{\ln[p + (p^2-1)^{1/2}]}, \qquad \text{(S7)}$$

For oblate:

$$\frac{f}{f_{\mathrm{sp}}} = \frac{(p^2-1)^{\frac{1}{2}}}{p^{\frac{2}{3}}\tan^{-1}\left[(p^2-1)^{\frac{1}{2}}\right]}. \qquad \text{(S8)}$$

Thus, p is calculated from f/f_0, which is experimentally obtained using the AUC. For a particle whose structure is unknown, it is difficult to judge whether it should be assumed to be prolate or oblate. Here, there is only a 3% difference in f/f_{sp} between prolate and oblate at $p \leq 6$: this difference is much smaller than the typical experimental error of f/f_0. Therefore, there is no significant difference in the finally obtained p even if either equation, Eq. (S7) or (S8) is used, except for the case with an extremely large asymmetry.

§7. Application to SANS data (AUC-SANS)

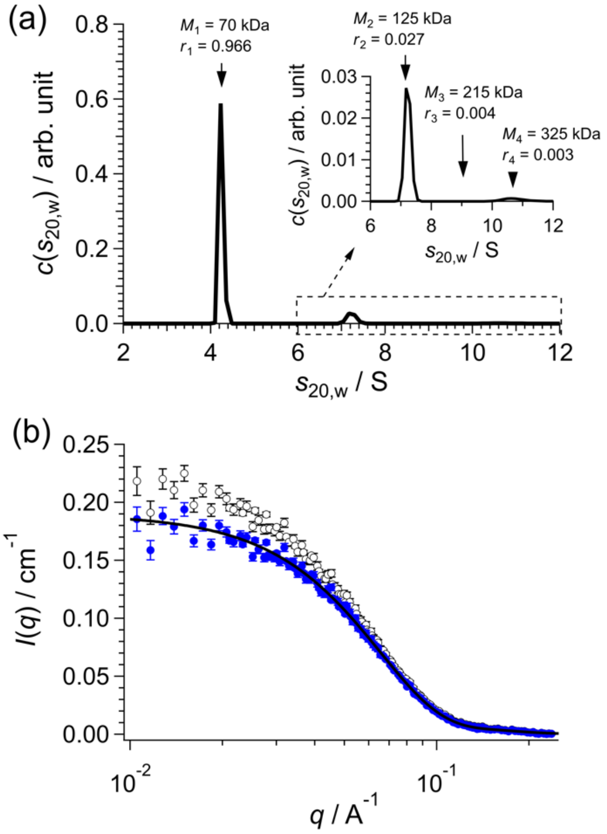

Figure S4. (a) Weight concentration distribution, $c(s_{20,\mathrm{w}})$, obtained with AUC for BSA3. Molecular weights M_j and weight fractions r_j for component j are shown in the figure. Frictional ratio f/f_0 was 1.42. (b) Open black circles show the concentration-normalized scattering profile, $i(q)$, given with SANS measurement for BSA3. Closed blue circles show the concentration-normalized scattering profile of BSA monomer, $i_1(q)$, derived with improved-AUC-SANS. Solid black line shows $i_1(q)_{\mathrm{Xtal}}$ calculated from the crystal structure.

Table S2. Gyration radii and forward scattering intensities for BSA3

	R_{g}, R_{g1}/ Å	$i(0), i_1(0)$ / $\mathrm{mg}^{-1}\mathrm{cm}^2$
Non-treated-SANS (BSA3)	27.0 ± 0.2	0.0531 ± 0.0008
Improved-AUC-SANS (BSA3)	26.5 ± 0.2	0.0512 ± 0.0008
Crystal structure (PDB code: 4F5S)	26.7	0.0585

R_{g}: gyration radius of untreated SANS. R_{g1}: gyration radius of monomer derived from improved AUC-SANS. $i(0)$: concentration-normalized forward-scattering intensity for untreated SANS. $i_1(0)$: concentration-normalized forward scattering intensity derived from AUC-SANS.

§8. The error originated by the random flight model.

The error originated by using random flight model was evaluated. Here, we consider the random flight model and two extreme models (linearly aligned and closed packing models). Figure S5 shows the inter-subunit structure factors $T_j(q)$ of random flight (RF), linearly aligned (lin), and closed packing (pac) models at j = 3 (a) and 4 (b). ($T_j(q)$ for random-flight model was calculated with Eq.(10), and those for linear aligned and closed packing arrangements are described in the previous paper; Morishima *et al.*, 2020). Subsequently, the scattering profiles of a monomer for each model ($I_{1,\mathrm{RF}}(q)$, $I_{1,\mathrm{lin}}(q)$, and $I_{1,\mathrm{pac}}(q)$) were calculated with Eq.(15). (Here, $\beta(q)$ was calculated with f/f_0 = 1.4 and Eqs.(9), (11)-(14), and (S4)-(S6).) Figure S5(b) shows the $I_{1,\mathrm{lin}}(q)/I_{1,\mathrm{RF}}(q)$ and $I_{1,\mathrm{pac}}(q)/I_{1,\mathrm{RF}}(q)$ in the cases of (r_1, r_4) = (0.8, 0.2). We note that the deviations of $I_{1,\mathrm{lin}}(q)/I_{1,\mathrm{RF}}(q)$ and $I_{1,\mathrm{pac}}(q)/I_{1,\mathrm{RF}}(q)$ from unity in $qR_{\mathrm{g1}} \geq 1.3$ indicate the maximum errors under the prerequisite condition ($r_\mathrm{a} \leq 0.2$ and $j \leq 4$). The maximum deviations of $I_{1,\mathrm{lin}}(q)/I_{1,\mathrm{RF}}(q)$ and $I_{1,\mathrm{pac}}(q)/I_{1,\mathrm{RF}}(q)$ were calculated to be 2.2 % (at qR_{g1} = 2.7; magenta arrow in Figure S5(b)) and 4.5 % (at qR_{g1} = 2.2; cyan arrow in Figure S5(b)), respectively. In conclusion, even if the random-flight model is applied to AUC-SAS, the error of the scattering intensity is a several % at most under the condition of r_a $\leq$ 0.2 and $j \leq 4$.

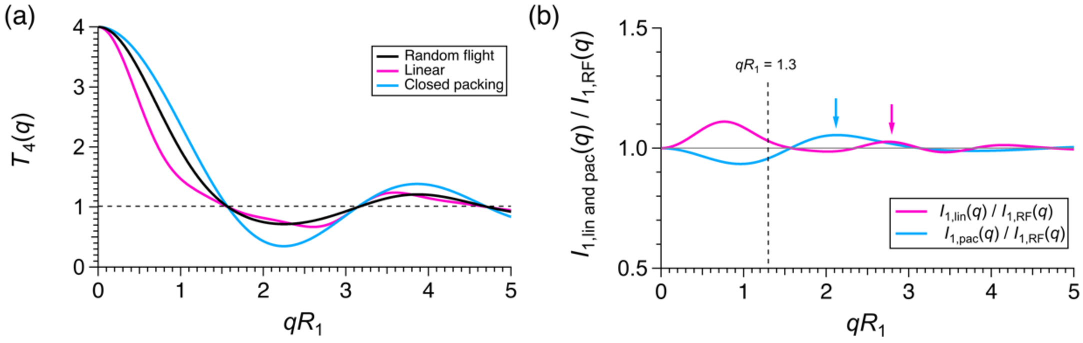

Figure S5. (a) Inter-subunit structure factor $T_j(q)$ at j = 4. Black, magenta, and cyan curves represent the $T_4(q)$ calculated with the random flight, linear aligned, and closed packing models, respectively. (b) Scattering intensity ratios, $I_{1,\mathrm{lin}}(q)/I_{1,\mathrm{RF}}(q)$ (magenta) and $I_{1,\mathrm{pac}}(q)/I_{1,\mathrm{RF}}(q)$ (cyan), in the cases of (r_1, r_4) = (0.8, 0.2). Magenta and cyan arrows represent the point where the deviations are maximum.

§9. The error originated by the ellipsoidal approximation.

To evaluate the error originated by ellipsoidal approximation to the improved-AUC-SAS, the shape anisotropy factor $\beta(q)$ was calculated with Eq.(9) ($\beta(q) = |\langle F(\boldsymbol{q})\rangle|^2/\langle|F(\boldsymbol{q})|^2\rangle$) via the following two ways: (i) $\beta(q)$ was obtained using the ellipsoidal $F(\boldsymbol{q})$ with frictional ratio f/f_0. (ii) $\beta(q)$ was obtained with $F(\boldsymbol{q})$ calculated from a crystal structure. Next, the scattering profile of a monomer $I_{1,\mathrm{el}}(q)$ and $I_{1,\mathrm{Xtal}}(q)$ were calculated with Eq.(15) using $\beta(q)$ via (i) and (ii), respectively. Figure S6 shows the $I_{1,\mathrm{Xtal}}(q)/ I_{1,\mathrm{el}}(q)$ for BSA (f/f_0 = 1.39), apoferritin (f/f_0 = 1.26), and Lysozyme (f/f_0 = 1.21), and βB2-crystallin (f/f_0 = 1.37) in the case of (r_1, r_4) = (0.8, 0.2). Here, we note the deviation of $I_{1,\mathrm{Xtal}}(q)/ I_{1,\mathrm{el}}(q)$ from unity in $qR_{\mathrm{g1}} \geq 1.3$ indicates the maximum error caused from ellipsoidal approximation under the prerequisite condition ($r_\mathrm{a} \leq 0.2$ and $j \leq 4$). For these proteins, the deviation of $I_{1,\mathrm{el}}(q)$ from $I_{1,\mathrm{Xtal}}(q)$ is 1% at most throughout $qR_{\mathrm{g1}} \geq 1.3$.

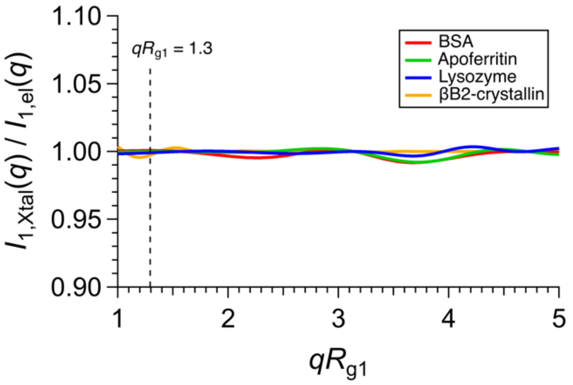

Figure S6. Red, green, blue, yellow lines show the scattering intensity ratios, $I_{1,\mathrm{Xtal}}(q)/I_{1,\mathrm{el}}(q)$ in the case of (r_1, r_4) = (0.8, 0.2) for BSA, apoferritin, lysozyme, and βB2-crystallin, respectively.

§10. AUC-SAS software.

The Igor Pro-based software for AUC-SAS (improved-AUC-SAS) is available at [http://www.rri.kyoto-u.ac.jp/NSBNG/activity.html] (a test dataset for BSA20 is attached). This software was developed with Igor Pro version 8.04, and is executable on Igor Pro 6, 7, 8, 9, and their free demonstration versions (https://www.wavemetrics.com).

Usage:

1. Download and open the Igor Pro file (AUCSASv4.pxp) at [http://www.rri.kyoto-u.ac.jp/NSBNG/activity.html].
2. Open the AUC-SAS panel (Figure S7) via the menu-tab (AUCSAS > Start AUCSAS).
3. Load the experimental SAS data with [Select file] button. The data file should be in tab-delimited text format (*. txt, *.dat, or *.csv) that consists of q, $I(q)$, and the errors of $I(q)$. We then input the available minimum q value into the SAS data.
4. Load the experimental AUC data with [Select file] button. The data file should be in tab-delimited text format (*. txt, *.dat, or *.csv), which consists of molecular weights $\{M_j\}$ and weight fractions $\{r_j\}$ for all the components obtained from $c(s_{20,w})$. Then, the frictional ratio f/f_0 (default value of 1.40) is input.
5. Push the [Start AUC-SAS] button after filling the output name. If the result is saved as. txt/.jpg files, put a check mark. Here, the output text file named [output name]_AUCSAS.txt offers q, $c_1 i_1(q)$, and the errors of $c_1 i_1(q)$. The structural parameters (R_{g1}, $c_1 i_1(0)$, and q_c) are also output as other text files ([output name]_Rg_I0.txt and [output name]_parameters.txt).

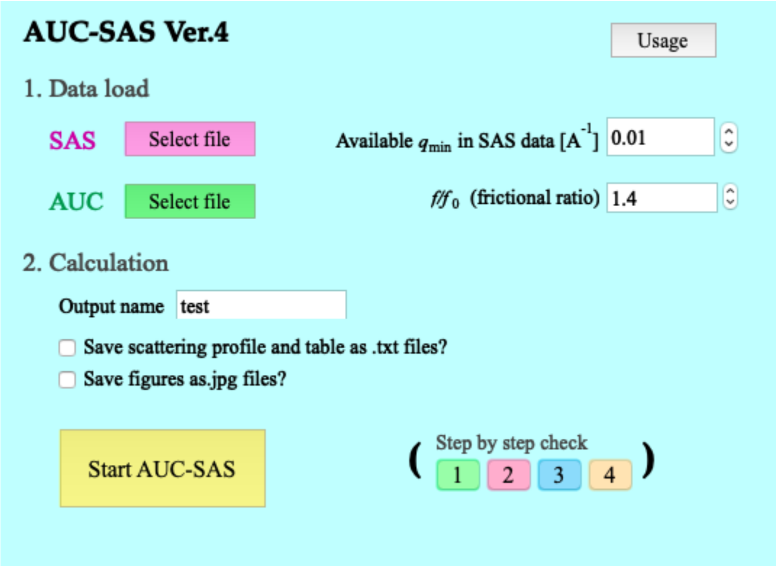

Figure S7. AUC-SAS panel on the Igor Pro software.

REFERENCES

Inoue, R., Nakagawa, T., Morishima, K., Sato, N., Okuda, A., Urade, R., Yogo, R., Yanaka, S., Yagi-Utsumi, M., Kato, K., Omoto, K., Ito, K. & Sugiyama, M. (2019). *Sci. Rep.* **9**, 12610.

Kotlarchyk, M. & Chen, S. H. (1983). *J. Chem. Phys.* **79**, 2461-2469.

Lebowitz, J., Lewis, M. S. & Schuck, P. (2002). *Protein Sci.* **11**, 2067-2079.

Morishima, K., Okuda, A., Inoue, R., Sato, N., Miyamoto, Y., Urade, R., Yagi-Utsumi, M., Kato, K., Hirano, R., Kujirai, T., Kurumizaka, H. & Sugiyama, M. (2020). *Commun. Biol.* **3**, 294.

Perkins, S. J. (2001). *Biophys. Chem.* **93**, 129-139.

Perrin, F. (1934). *J. Phys. Radium* **5**, 497.

Pierce, B. G., Wiehe, K., Hwang, H., Kim, B.-H., Vreven, T. & Weng, Z. (2014). *Bioinformatics* **30**, 1771-1773.
